# Unsupervised Human Detection with an Embedded Vision System on a Fully Autonomous UAV for Search and Rescue Operations

**DOI:** 10.3390/s19163542

**Published:** 2019-08-14

**Authors:** Eleftherios Lygouras, Nicholas Santavas, Anastasios Taitzoglou, Konstantinos Tarchanidis, Athanasios Mitropoulos, Antonios Gasteratos

**Affiliations:** 1 Department of Production and Management Engineering, Democritus University of Thrace, 67100 Xanthi, Greece; 2 Department of Petroleum, Natural Gas and Mechanical Engineering, Eastern Macedonia and Thrace Institute of Technology, 65404 Kavala, Greece

**Keywords:** unmanned aerial vehicles (UAVs), search and rescue (SAR) missions, human detection, deep learning

## Abstract

Unmanned aerial vehicles (UAVs) play a primary role in a plethora of technical and scientific fields owing to their wide range of applications. In particular, the provision of emergency services during the occurrence of a crisis event is a vital application domain where such aerial robots can contribute, sending out valuable assistance to both distressed humans and rescue teams. Bearing in mind that time constraints constitute a crucial parameter in search and rescue (SAR) missions, the punctual and precise detection of humans in peril is of paramount importance. The paper in hand deals with real-time human detection onboard a fully autonomous rescue UAV. Using deep learning techniques, the implemented embedded system was capable of detecting open water swimmers. This allowed the UAV to provide assistance accurately in a fully unsupervised manner, thus enhancing first responder operational capabilities. The novelty of the proposed system is the combination of global navigation satellite system (GNSS) techniques and computer vision algorithms for both precise human detection and rescue apparatus release. Details about hardware configuration as well as the system’s performance evaluation are fully discussed.

## 1. Introduction

UAV technology demonstrates unprecedented levels of growth and influence in various scientific fields and research areas. Owing to their tremendous advantage of being able to automatically perform complex tasks and missions, the application domains of such vehicles are ceaselessly increasing. One of the most challenging application areas of such aerial robots is SAR missions, as crisis events may occur at any given moment and over all kinds of places [1]. The success rate of such procedures is highly depended on the time needed for first responder awareness that a human life is in imminent danger. The faster these operations are designed and executed, the greater the chances of success they get. Thus, this application area can greatly benefit from aerial robots capable of detecting and rescuing humans facing dire straits in, mainly but not only, maritime critical events [1,2,3,4]. Rescue UAVs are valuable during crisis management due to their maneuverability and versatility. These vehicles are capable of speeding up rescue missions while improving their efficacy. Additionally, with a view to increase the victim’s survival time, these flying robots can transport rescue apparatus much faster than rescue crews [5]. Moreover, such vehicles should be plausibly considered as a robust helping hand to the first responder teams as they are beneficial in terms of providing aerial visibility of the critical event while ensuring rescue worker safety [6].

The utilization of UAVs as a complement of search and rescue activities has gotten continuously increasing support by researchers and organizations around the globe. Numerous rescue systems including teleoperated UAVs have been proposed to establish robotic assistance in emergency management [7,8]. Taking into account the emerging necessity for an utterly autonomous rescue UAV, an aerial rescue support system was proposed in our prior research [9,10]. With the aim to navigate and operate fully autonomous ROLFERs (Robotic Lifeguard For Emergency Rescue), our aspiration is to provide instantaneous emergency services to humans in peril during a maritime crisis occurrence while boosting rescue team performance by significantly minimizing their workload. Detecting distressed humans from the developed aerial rescue system has mainly relied on GNSS techniques. Notable research has been conducted for the GNSS precise point positioning (PPP) accuracy of portable devices such as smartphones, tested in both post-processing and real-time modes [11,12,13]. The results of their positioning performance assessment compared to the geodetic receivers’ ones indicate that the utilization of multi GNSS systems data can effectively improve positioning performance, which is essential for missions in marine environments. Besides, due to the lack of commercially available wearable, waterproof, and portable devices with acceptable accuracy, there is a pressing need to enhance the system’s operational performance through the precise location of the victim. The obvious solution to employ computer vision techniques to solve this problem is not straightforward, owing to several restrictions in conventional classifier accuracy and precision. That have been said, contemporary deep learning techniques [14] can overcome such issues, yet their complexity makes their implementation on a portable system, where hardware means should be rather austere, a cumbersome procedure.

The paper in hand proposes an embedded system implementing a deep learning architecture trained for the identification and location of swimmers. Thus, we address the problem of precise open water human detection by conducting real-time recognition onboard a rescue hexacopter, as well as the accurate transportation and release of the airborne rescue apparatus. The main novelty of this research banks on the collection of a novel dataset, suitable for training state-of-the-art computer vision algorithms, the implementation of deep learning algorithms on an embedded system suitable for a medium payload carrying capability hexacopter, and their seamless integration with an utterly autonomous search and rescue system. In particular, in order to obtain an adequate size dataset for the chosen model, the majority of the training dataset used in this research are images extracted by videos captured by the authors. Humans swimming in open waters were filmed by means of a UAV flying at several altitudes. The annotated dataset is freely available to any member of the community. The remainder of this paper is structured in the following manner. Section 2 provides an overview of the conducted related research. Theory and approach of our work are presented in Section 3. The system’s human detection procedure is described in Section 4. In Section 5 we provide some crucial information about the hardware configuration, as well as the system’s implementation details. Conclusions are drawn in Section 6.

## 2. Related Research

Object detection has been a broad field of study in computer vision over the past decades. The superior performance of deep learning techniques in detecting and classifying objects in images has led to their widespread adoption in a wide variety of applications [15]. Several approaches and architecture for object detection, segmentation and action recognition throughout videos have been proposed in the literature, including many variations of convolutional neural networks (CNNs) such as temporal convolutional networks (T-CNN) [16,17,18], 3D CNNs [19,20,21] and many more. 

A noteworthy overview of deep learning methods and applications combined with UAVs technology has been conducted in [22]. The main challenges, as well as the performance and limitations of such developments were discussed in detail. Furthermore, several unsolved challenges for efficient performance of unsupervised learning were pointed out. Yet, the selection and the adjustment of the operational parameters for the training of CNNs capable of performing object detection are of vital importance due to the high dependency of a model’s reliability and accuracy to such procedures [23]. An approach for the implementation of such systems has been presented in [24]. Data collection, deep networks architecture and training stages for an embedded processing platform, performing vehicle detections through aerial images, are highlighted. 

Bearing in mind UAV competence in providing precious aerial visual information, the implementation of such techniques on aerial systems is an increasing trend in various research fields [25,26,27,28,29]. Issues related to sensing and connectivity of sensors for the assisted or even the autonomous navigation of UAVs, as well as the reliability and the maintenance analysis of such vehicles have been extensively discussed in [30,31]. Moreover, the adaption of camera parameters, as well as motion parameters estimation based on the integration of video measurements and data obtained by UAVs, have always to be seriously taken into account [32]. The relentless motivation for image classification tasks based on deep learning and aerial images captured by UAVs has led to an abundance of research including vehicles [24,33,34], aerial vehicles [35,36,37,38,39], roads [40], buildings [41,42], cracks [43], birds [44], cattle [45], and wilt [46] detection. Another remarkable approach for object detection in very high-resolution aerial images has been proposed in [47]. Using a one-stage densely connected feature pyramid network, the proposed model tackles the problem of target object detection on different scales. Assessment on two datasets in terms of computation time and mean average precision (mAP) was also provided. A primordial task for researchers dealing with computer vision and UAVs, is the precise autonomous landing of such vehicles on both static or moving targets [48,49,50,51]. The detection of a desired target and the landing of the aerial vehicles on a desired target totally depend on onboard sensing and computing, without requiring external infrastructure or prior information of the target’s location. Nevertheless, human identification in videos or images is the most crucial task across a variety of scientific fields. Thus, real-time human detection from an aerial perspective based on deep learning approaches is an extremely active research area [52,53,54]. Consequently, when it comes to crisis events, a dramatic demand for rapid and accurate detection of an individual whose life is at risk arises, as the provision of instantaneous rescue services is urgent for his/her survival. 

Search and rescue operations can greatly benefit by deep learning techniques applied in UAVs, as such developments exhibit high performance in detecting and precisely locating humans in peril while decreasing the response time that rescuers would need to do so. Nevertheless, the separation of crowded and non-crowded regions for safe UAV flight is apparent. The research in [55] proposes a novel method for enhancing the definition of non-fly zones for crowd avoidance by utilizing deep CNNs. A notable approach for the detection of human survivors in disaster environments has been conducted in [56]. The proposed system detects human existence by applying a feed-forward neural network, acquiring a small amount of data with high detection accuracy. In [57] a method for SAR mission assistance in case of avalanche debris was proposed. Furthermore, an innovative, state-of-the-art technique for the estimation of snow-field parameters has been presented in [58]. The unmanned aerial system (UAS)-based photogrammetry method significantly reduces the data collection time and health risks associated with avalanches. A system composed of a pre-processing method, a pre-trained CNN, a trained linear support vector machine (SVM) classifier and a post-processing method for human detection rate increase in images captured by a UAV, is analytically presented there. The performance of accurate real-time object detection on-board a UAV has been outlined in [59]. Capable of accurately detecting objects during emergency situations, the developed system critically feeds a decision support warning system for situational awareness. In [60], a notable approach for victim detection in complex environments based on the color and depth data captured from onboard UAV sensors was proposed. The implemented reactive control system’s validation, including both real flights and simulations, exhibited interesting results about multiple individuals tracking even after long-term disappearances. Last but not least, a project for industrial firefighter performance enhancement has been presented in [61]. Aiming to reduce their cognitive load, firefighters can benefit from mission-guided control and enhanced autonomy of the system in known indoor and outdoor environments by assigning mission goals with priorities to drones.

In conclusion, advances in deep learning have attracted researchers’ interest for applying deep reinforcement learning methods to rescue robots for their autonomous navigation [62], efficient exploration of various unknown environments [63], SAR purposes in indoor environments [64], and for target recognition and interaction between aerial robots and targets.

## 3. Deep Learning

Deep learning methods and techniques have been getting a dramatically increased popularity among the research areas of neural networks and artificial intelligence. Their increased processing capabilities, the huge amount of data used for training, and the recent advances in machine learning and signal processing are the main reasons for being held in high regard by researchers [15]. Their superior capabilities in solving complex tasks on many facets of information processing have led to their breathtaking impact on numerous application areas. Autonomous robotics applications are a bold example of the suitability of deep learning techniques utility and their huge beneficial potential. The combined use of such methods and UAV technologies have presented a drastic increase recently, due to the fact that such techniques are capable of extracting exceptionally useful information from aerial images [22]. Convolutional neural networks are state-of-the-art in the advanced application of deep learning for a broad range of tasks such as image or video processing, speech recognition, text digitization, and more. CNNs utilization for image-related purposes is the most popular application domain of this specific type of neural network algorithm. Their marvelous capability of detecting what an image is or what an image contains, or even automatically generating captions for images, has impressively increased their popularity and their feasibility in the computer vision field [65,66].

YOLO [67,68] is an open source state-of-the-art object detection system for real-time processing. Using a totally different approach, YOLO has several advantages, compared to prior region object detection systems and classification networks, in the way it performs detection and prediction. Region proposal classification networks perform detection by applying the model to an image with multiple predictions in various image regions and scales. High scoring regions are considered as detections, yet YOLO uses a one-stage detector strategy and its architecture is similar to a fully convolutional neural network (FCNN). Specifically, YOLO treats object detection as a regression problem by applying a single CNN and bounding boxes to the full image, for both the object’s classification and localization. The image is divided into regions and predictions are made by bounding boxes weighted by the predicted probabilities. The main YOLO advantage for real-time object detection is the improvement of deep learning-based detection procedure speed. 

## 4. Human Detection

Detecting humans in images cropped from a video stream either on land or a marine environment, is a challenging task due to the human body’s variable appearance and the wide range of poses it can adopt. Moreover, video footage collected by a UAV differs substantially from images acquired on the ground; therefore the use of standard techniques is not straightforward. The following aspects and assumptions must be considered when designing the image processing algorithm. Firstly, the typical distances from a camera to human bodies of interest are generally larger than in the case of standard “ground” techniques (e.g., office-like environments, surveillance cameras). Additionally, the human footage in a marine environment may differ substantially from a land one. In a common land situation, the whole human silhouette should be visible, yet in a water one it is most likely that only a part of it (i.e., the upper body) is visible. Moreover, should the vehicle be operational during different times of the day, the human detection algorithm must be equally effective under all lighting conditions. Therefore, it is essential for the image processing algorithm to take into account all the aforementioned considerations. A robust feature set that allows the human silhouette to be discriminated clearly, even in cluttered backgrounds under different poses and different illumination conditions, is apparently needed. In this article we study the combination of the open source software routines, libraries and algorithms available today with a low cost vision camera, in order to design a system that can effectively detect, localize, and track a human in a marine environment, under all lighting conditions. For this reason, we focus on computational efficient computer vision algorithms; even at the cost of low frame rate.

**Figure 1:**
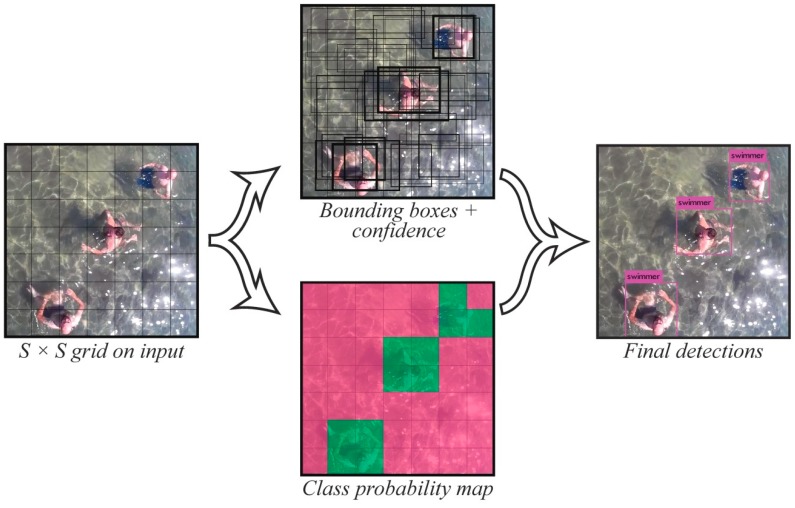
Image captured by the rescue unmanned aerial vehicle (UAV): (Left) divided into equally *S* × *S* sized grid cells, (Top and Bottom) predicting *B* bounding boxes, confidence scores and *C* conditional class probabilities, (Right) leading to final encoded detections.

In order to detect swimmers, each frame of live video captured by the camera onboard the UAV is processed separately by the pipeline. Firstly, the input image is divided into a *S* × *S* grid with *N* number of cells, as shown in Figure 1 (Left). Should the centre of the object be laid within a grid cell then this specific cell is “responsible” for the object’s detection. At this point, each grid cell predicts *B* bounding boxes as well as confidence scores for those boxes (see Figure 1 (Top)). These confidence scores reflect the confidence of the model that an object is contained in the box, as well as the accuracy of the box that it predicts. Confidence is formally defined as

(1) $$Conf(\%)=(P_{r}(object)\times IoU_{pred}^{truth})\times 100\%$$

where *Conf* is the confidence level expressed in a percentage score, *P_r_* is the predicted box containing an object, and *IoU* is the intersection over union between the predicted box and the ground truth. 

In the ideal situation where the object is contained within the predicted box, then *Conf* equals *IoU*; while should no object exist in the box, *Conf* equals zero. 

Every single bounding box contains 5 predictions, namely: The (*x*,*y*) coordinates of the bounding box center, the box dimensions (i.e., width (*w*) and height (*h*)) and finally the probability of objects’ appearance in the bounding box (*P_r_*(*object*)) [67,68]. All these are normalized values (i.e., between 0 and 1). Bounding box center coordinates (*x*,*y*) are calculated relatively to the bounds of grid’s cell, while the width and the height are predicted relatively to the whole image. Thus, the confidence prediction expresses the *IoU* factor between the ground truth and the prediction box.

Moreover, each grid cell predicts *C* conditional class probabilities (*P_r_*(*ClassijObject*)), which are affected by the grid cell containing an object (see Figure 1 (Bottom)). Previous YOLO versions apply a softmax function to convert scores into probabilities with sum equal to 1. Instead, YOLOv3 [69] uses multi-label classification by replacing the softmax function with independent logistic classifiers in order to calculate the probability of an input belonging to a specific label. Thus, the model makes multiple predictions across different scales, with higher accuracy, regardless of the predicted object’s size. Finally, the network consists of two main parts—the base model, which operates as feature extractor and the resulting tensor, which encodes the bounding box, the probability of object’s existence in the cell (objectness), and the class predictions. The final output of the whole procedure (Figure 1 (Right)) is an encoded *S* × *S* × (B*5 + *C*) tensor where:

*S* × *S*: is the sized grid cells dimensions
*B*: is the number of bounding boxes and
*C*: is the number of networks’ labeled classes

During the training process, the loss function of end-to-end model should simultaneously address both classification and detection issues, which dictates the learning and the testing accuracy of the detectors. In YOLO, the computation of each prediction’s loss is accomplished by using the sum-squared error between the ground truth and the predictions. Thus, the loss function consists of three individual losses:

- Classification Loss: Errors in the prediction’s accuracy
- Localization Loss: Errors between predicted boundary box and ground truth
- Confidence Loss: Errors in the object’s appearance in the box

The model’s architecture used in this paper is the Tiny YOLO V3 [69]. Comprising an improvement of YOLO, Tiny YOLO v3 treats detection somewhat differently by predicting boxes on two different scales while features are extracted from the base network. Its higher performance compared to YOLO was the main reason for its selection.

**Figure 2:**
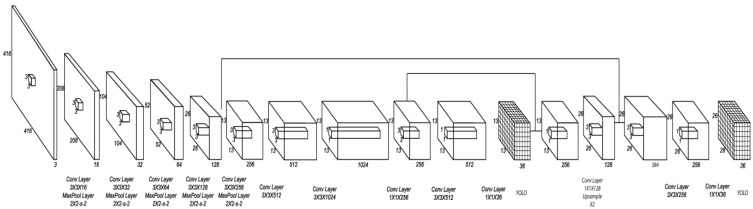
Convolutional neural networks (CNN) model’s architecture.

The model’s architecture consists of thirteen convolutional layers with an input size of 416 × 416 images, as shown in Figure 2. Due to the limited available amount of dataset, we used transfer-learning for a pre-trained model in order to train a CNN to detect swimmers. The model was trained using COCO dataset [70] and our “swimmers” dataset (https://robotics.pme.duth.gr/swimmers_dataset). The model’s training was accomplished by initializing and fixing the depth size of the last convolutional layer’s pre-trained weights. Then, we further trained the model with a hand-labelled dataset of open waters swimmers. The model’s achieved mAP was approximately 67%.

**Figure 3:**
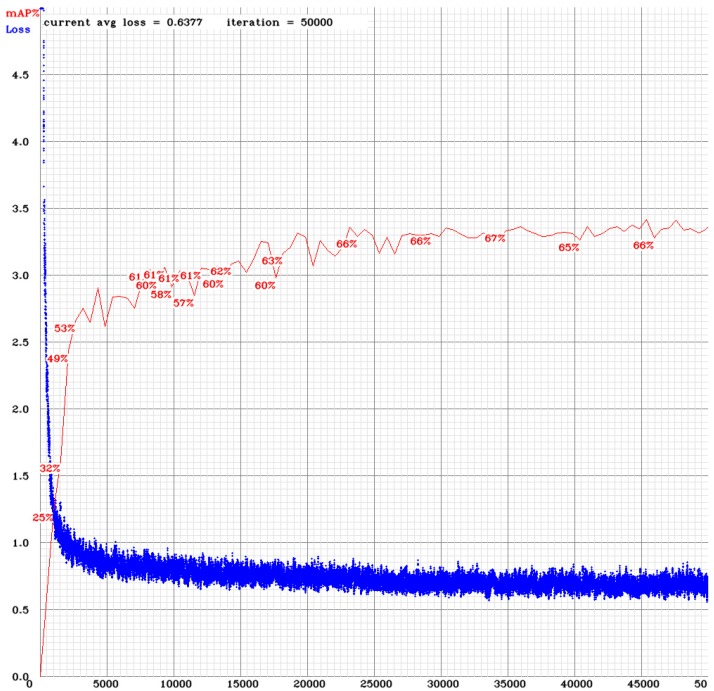
Neural Network’s training performance curve.

The relation of mAP to the iteration number of the utilized model is depicted in Figure 3. As it is shown, loss function rapidly decreased as iteration number increased. This provides a nice close view of all stages of the network’s learning process. Moreover, the classification accuracy on the test data also changes relatively to the number of iterations. As can be seen, in the first 3000 iterations, there is a big jump in the network’s accuracy to approximately 55%. Next, the accuracy gradually improves till 35,000 iterations, reaching 67%. Then, the learning process gradually slows down. Finally, at approximately iteration number IN = 44,000, the classification accuracy fairly stops improving. Later iterations merely see small stochastic fluctuations near the value of the accuracy at IN = 44,000. This means that the network ceases learning at this point, so we concluded that it was trained and after a number of iterations, started overfitting.

Concerning the training dataset, we collected from the internet as many swimmers’ images as possible, since there was no akin dataset available. Because of the low number of images collected, we were forced to create our own dataset, by manually flying a drone ashore and recording videos containing swimmers. Frames were recorded by means of a GoPro camera, in full HD resolution (1920 × 1080 pixels), and in different angles and lighting conditions. After the network’s first training attempt, we observed a significant number of false positive detections, such as boats. Thus, in order to deal with the low “recall” figures, we executed another run of image collection, in the same magnitude as the previously obtained images, yet containing objects that the network false detected. In this way we managed to increase recall and mAP. Eventually, a total of 4500 images were collected. For increased robustness and improved recall values, negative images at 1:1 ratio were also included. The total training dataset of the proposed CNN model was 9000 images, while 10% of them were kept as validation data. The dataset was split by 90% as training and 10% as validation dataset, i.e., the volume of the training set was 8100 images. All subjects were informed and gave their consent to conduct the experiments. The images gathered differed in terms of number of humans in the scene, human characteristics, image scales and background, lighting conditions, resolution, and composition. Finally, a total of 4500 images were collected. Last, with the aim to create our unique training dataset, all swimmer images had to be labeled. Thus, an open-source labelling tool was used in order to create ground truth bounding boxes in each of these images.

**Figure 4:**
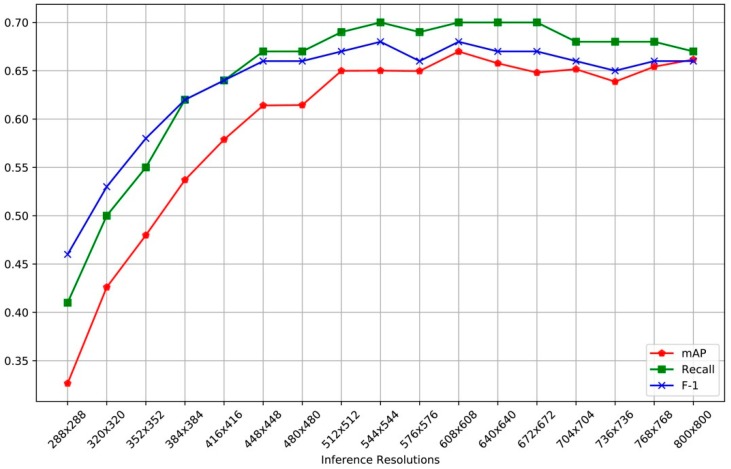
Metrics results for different input resolutions.

Although the video recording resolution was full HD, the network was multi-scaled trained. Because of the architecture’s nature, which was fully convolutional, every layer could be resized on the fly. During the training procedure, the model randomly downsampled the initial resolution (we chose 608 × 608) of every 10 batches by a factor of 32. This regime forces the network to learn to predict across a variety of input dimensions [68]. In the stage of inference, the network’s input resolution was set to 416 × 416 pixels and the results of mAP were calculated using this resolution. The YOLO algorithm resizes the training dataset to the initial resolution, as mentioned above. Due to the multi-scale training procedure, there is no need to change the initial resolution during training. The only resolution change that matters is the one along inference, because of the different performance results as depicted in Figure 4.

Throughout training procedure, the highest model performance was attained with the following network parameter values: Batch size = 64, momentum = 0.9, decay = 0.0005, learning rate = 0.001, threshold = 0.25, and iteration number = 45,000. The detector was able to recognize “swimmers” objects in the dataset with 67% mAP and 70% recall. Given these figures, the detector might seem unreliable in the short term and between consecutive frames, something that does not affect the sought application. With the aim to support our arguments, we provide a link (https://youtu.be/xxAaadmI5GA) containing experimental results from data never experienced by the network during training. 

**Figure 5:**
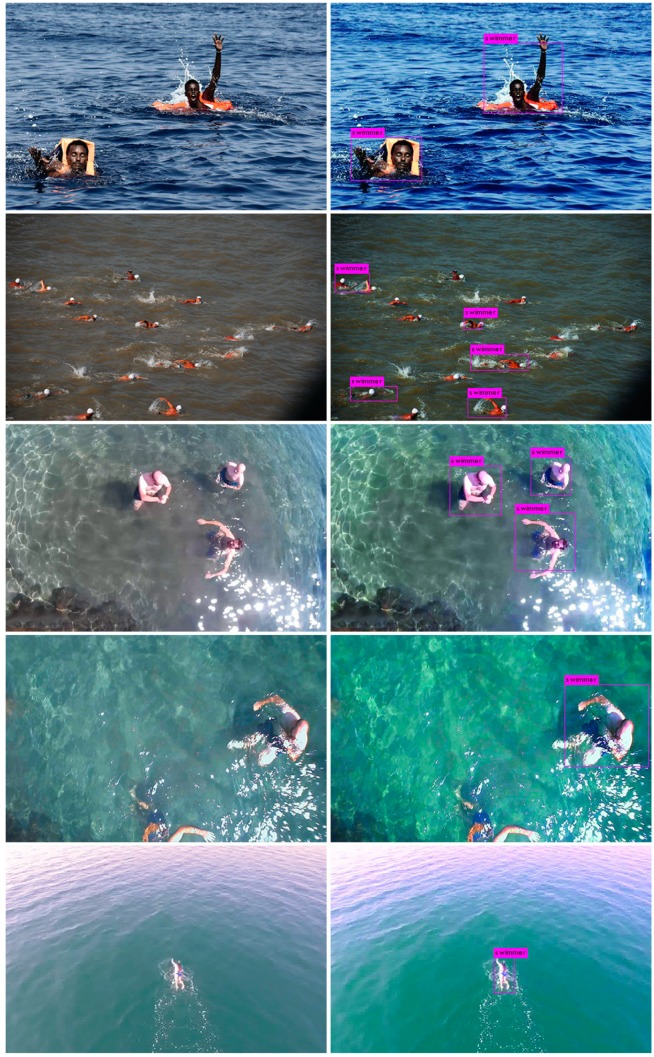
Humans in open waters (left column) before and (right column) after detection procedure.

The whole training procedure lasted almost a six-hour run on a Nvidia Tesla V100. The model was validated by assessing the classification accuracy on a class of objects, namely “swimmers”. This class consisted of images including open water swimmers (see Figure 5 (left column). Next, once validation and training have been completed, open water swimmer detection tests were performed on real-time videos captured by the rescue UAV, in order to evaluate network accuracy. Results from network testing are as shown in Figure 5 (right column). The model was able to accurately detect and classify the presence of human bodies or shapes in open water.

In the present work, a Darknet [71] framework was adopted for the detection of both landing targets and humans in peril. Darknet is an open source neural network framework which supports central processing unit (CPU) and graphics processing unit (GPU) computations suitable for a wide variety of tasks relative to computer vision. Darknet is written in C and CUDA and can be easily installed and applied for such tasks due to its extremely large computational power. The main reason for this selection was in order to achieve high-level accuracy as the whole detection procedure had to be implemented with an adequate frame rate due to a swimmer’s potential continuous movement. Darknet combined with YOLO provided adequate results due to its high-detection speed and low computation resources for our embedded system.

As an alternative to YOLO, we trained another detector, based on SSD MobileNetV2 [72] architecture with an input image resolution of 300 × 300. Using the same training dataset, we used transfer-learning by applying pre-trained weights from the COCO dataset to the model. However, this approach barely provided a mAP of 21%. Thus, it is apparent that YOLO outperforms the MobileNet approach for the given application, so we chose it.

## 5. Implementation on the Autonomous UAV

**Figure 6:**
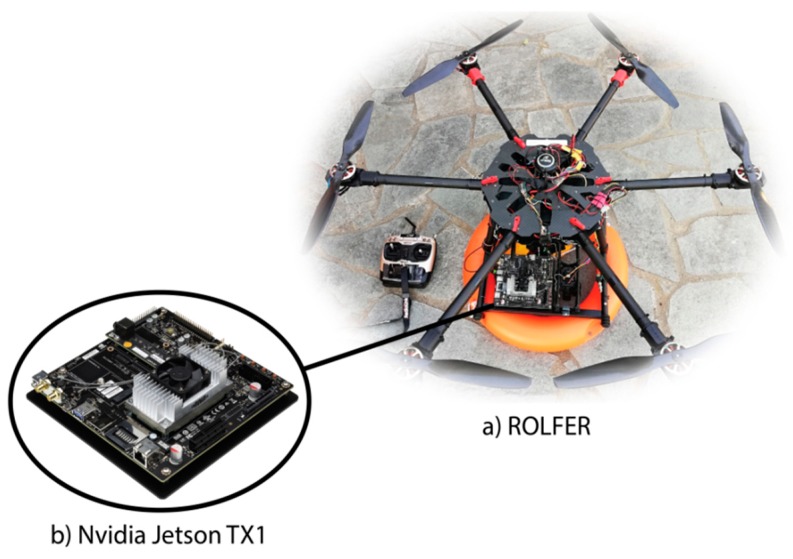
(**a**) The fully autonomous rescue UAV and (**b**) the embedded graphics processing unit (GPU) platform Nvidia Jetson TX1.

The primary objective of this project is the integration of machine learning methods to a fully autonomous rescue UAV, for real-time detection of humans in peril, in case of a maritime crisis event occurrence. Towards this end, the first step of this implementation was the application of a vision-based neural network controller for the fully autonomous landing of the rescue UAV on static targets, which was implemented and assessed on a hexacopter bearing an embedded system affording independent take-off, navigation, and landing on a fixed target. The embedded system used for this assessment was a Raspberry Pi 3 and the neural networks’ acceleration package was the NNPACK, the combination that provided the adequate effectiveness and accuracy. However, a higher resolution as well as a higher frame rate is needed for the integration of the aforementioned methods to a rescue UAV, due to a swimmer’s potential continuous movement. For fully autonomous UAV navigation, as well as the automatic detection of human in peril, the detection algorithm should run onboard. Owing to the fact that YOLO calculations are based on a CUDA Toolkit, a powerful GPU embedded system with low consumption would be required. Therefore, for the implementation of the system in this paper, the Nvidia Jetson TX1 was chosen. The algorithm is executed with 12 fps, which forms an adequate swimmer’s position update rate for the rescue aerial vehicle’s prompt performance. The aerial rescue vehicle’s technical specifications, used for the implementation of this research, are presented in Table 1. The fully autonomous rescue UAV and the embedded system for the real-time swimmer detection are depicted in Figure 6.

**Figure 7:**
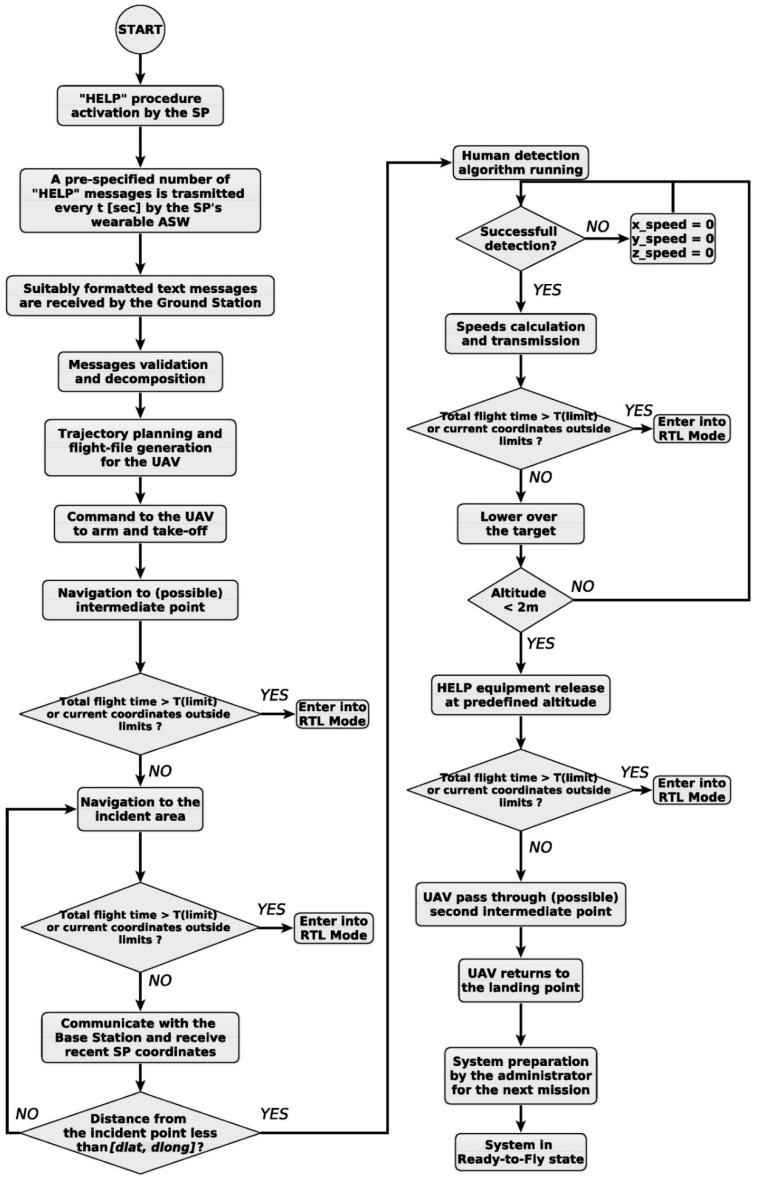
Autonomous Rescue System’s Operation Flowchart.

The entire rescue process of the proposed system, based on the image processing algorithm running on the on-board autonomous computation machine, is illustrated in Figure 7.

The rescue procedure is triggered by a “HELP” message submitted by the SP (Supervised Person—a distressed swimmer being in a supervised area in order to benefit from the proposed system) [9,10]. This message is received, validated and decomposed at the ground station (GS) by the main controlling computer. Then, the rescue aerial vehicle’s flight-file is generated. The UAV is commanded to automatically arm and take-off. It heads towards the incident area, possibly through one or more intermediate points in order to avoid potential obstacles or restricted regions [10]. Given the limitations of GNSS accuracy in commercially available Android smart watches (ASW) today, the UAV can reach the swimmer with an optimal horizontal position accuracy of ±5 m, with 95% confidence (also mentioned as 95th percentile). Owing to the complexity of the “GNSS accuracy” definition, a full-length documentary or a several page article would be appropriate to explain such a topic. The testing process for the accuracy of handheld GNSS is conducted under at least two main conditions: (a) an open sky, and (b) a disturbed atmosphere with plenty of obstructions, such as is an urban area with high skyscrapers, which is the worst-case scenario. As we are dealing with open water swimmers or other humans in peril, we assumed that we fall into the first category. Thus, we will mention only the maximum accuracy of GNSS.

**Figure 8:**
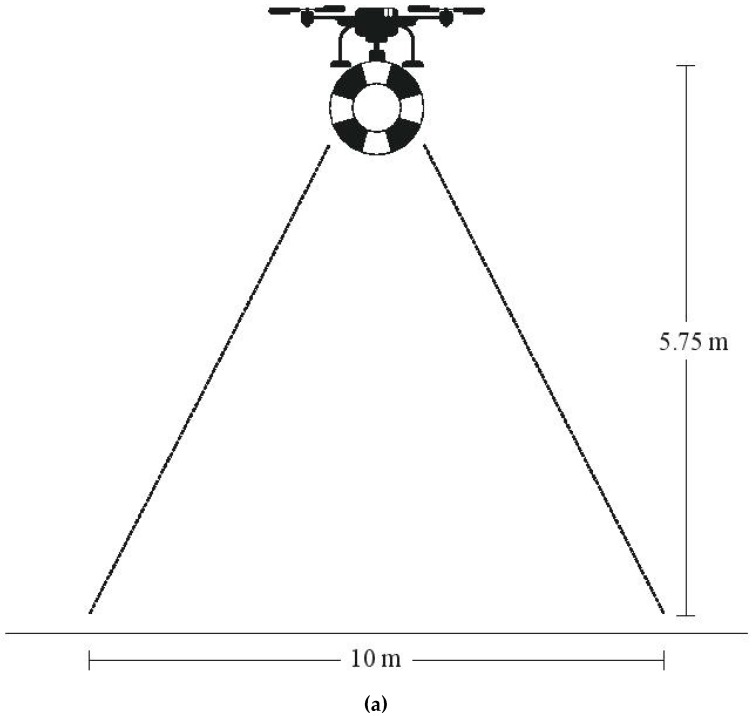

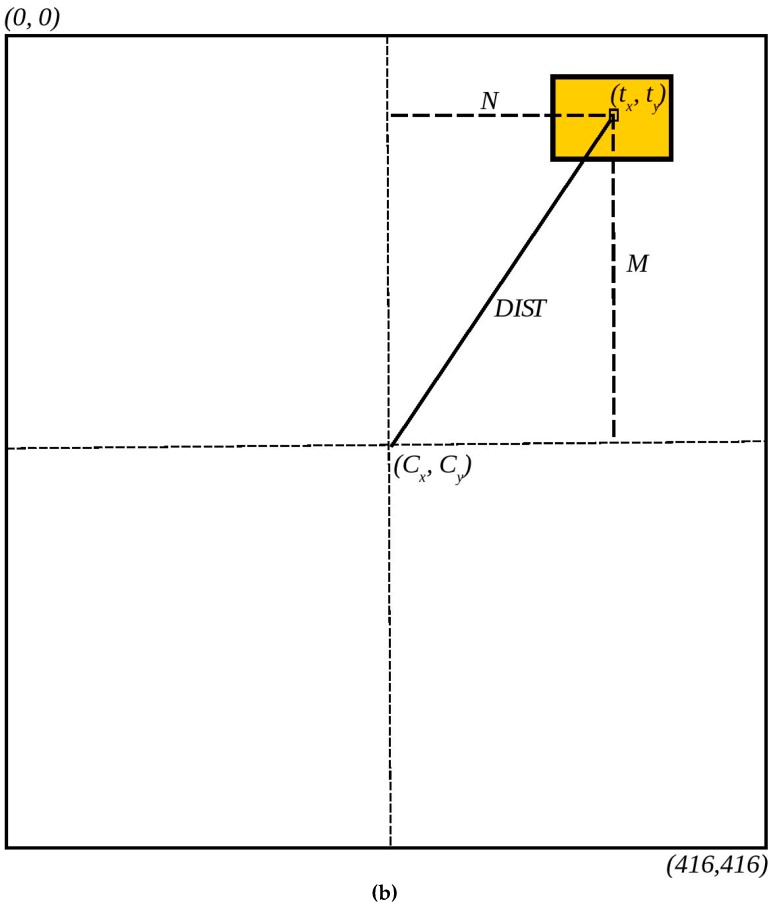
Rescue UAV’s camera field of view (FoV) (**a**) The proportion between sides *N*, *M* and (**b**) speeds *t_x_*, *t_y_* on *x*, *y* axes.

For the autonomous rescue UAV to reach the incident area, the UAV’s controller checks if the absolute distance between the swimmer and the UAV is less than the pre-specified limits (dlat, dlong) defined by the entire system’s administrator during its setup. Details about these limits are analytically described in [9,10]. At this point, the potential victim is located within the Field of View (FoV) of the onboard camera, while the UAV flies above a minimum altitude. Provided that our camera’s FoV is 82o, the minimum Altitude in order to cover the complete circle of radius R = 5 m is about Alt = 5.75 m (see Figure 8a). Since the UAV is flying at Alt = 10 m, the human in peril will certainly be located inside the cameras’ FoV. Then, image processing will be activated and the human detection algorithm will be activated on the on-board autonomous computing machine. A successful human detection process will return the distressed swimmer’s coordinates in the image frame and the control algorithm’s calculation for the UAV’s speed in *x*, *y* axes will be activated. Next, the main objective of the UAV motion controller is the aerial vehicle’s movement in the proper direction in order to be exactly over the swimmer and to start descending at a pre-specified altitude above him/her. The validation of the UAV’s precise positioning is a crucial factor in such rescue systems as the rescue apparatus should be released as close as possible to the distressed human, at a maximum distance of ±0.5 m.

The available data are the camera’s resolution and image coordinates of the two opposing angles of the orthogonal box in which the swimmer is located. Image coordinates are defined as the number of pixels of those two points, with axes origin placed at the top left angle (0, 0) and the opposing angle at 416 × 416 image resolution. The UAV’s center is defined by the central camera’s pixel to *x*, *y* axes correspondingly (see Figure 8b), due to the fact that the camera is placed in the middle of the box, i.e., at

$$C_{x}=\frac{416}{2},$$

$$C_{y}=\frac{416}{2}.$$

As mentioned above, the main objective is the aerial vehicle’s movement in the direction and to the center of the detected swimmer’s area. 

In order to control the vehicle’s speed in 3D space, the desired speed’s components *v_x_*, *v_y_*, *v_z_* on each of *x*, *y*, *z* axes, correspondingly, must be calculated and provided to the UAV’s controller. Since the *z*-axis speed is the descending speed to the target, it can be defined separately through the UAV control software.

If (*t_x_*, *t_y_*) are the coordinates of swimmer’s image bounding box centroid (see Figure 8b) and (*c_x_*, *c_y_*) are the UAV’s center coordinates, then sides *N*, *M* are known, since:

(2) $$N=t_{x}-C_{x}$$

(3) $$M=t_{y}-C_{y}$$

Thus

(4) $$dist=\sqrt{N^{2}+M^{2}}$$

From Equations (4)–(6), it is obvious that the direction of the vector of resultant speed is equal to the direction of the vector of the hypotenuse dist, resulting from the proportion ratio between the sides *N*, *M* and the speeds on *x*, *y* axes.

Given the proportionalities, we can further derive that:

(5) $$\frac{speed_{y}}{N}=\frac{total_{speed}}{dist}$$

(6) $$\frac{speed_{x}}{M}=\frac{total_{speed}}{dist}$$

where total_speed_ is equal to the defined maximum resultant speed. Thus, the resultant of the two individual speeds, directs the hexacopter towards the swimmer.

**Figure 9:**
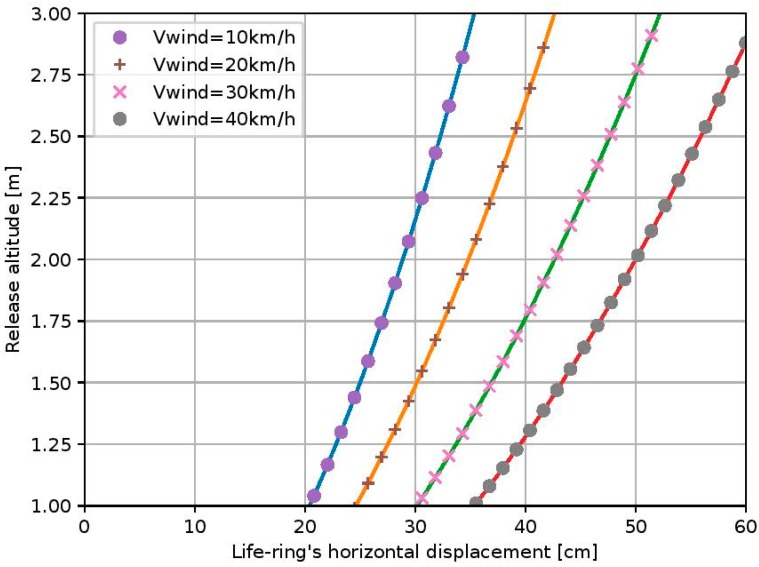
The life-ring’s horizontal displacement related to the UAV’s release altitude under several wind speeds.

Upon the precise detection of the distressed human, the autonomous rescue hexacopter descends at a system’s administrator-defined altitude above him/her and the rescue apparatus is released. After that action, the hexacopter returns to the shore. Figure 9 depicts the simulation results for the expected displacement of a released ring with R = 25 cm diameter and an elliptical intersection of 10 × 8 cm (a = 5x, b = 4), under the action of side-wind.

Results of the life-ring’s release accuracy were calculated after simulating the whole rescue apparatus release process. Air flow velocity is considered as a random variable. Bearing in mind that air resistance is proportional to the square of falling velocity, the life-ring’s equation of motion can be expressed as

(7) $$m\frac{dv}{dt}=mg-\frac{1}{2}\rho C_{d}Av^{2}$$

where:

*m* is the life-ring’s mass (*Kg*),
*A* is the life-ring’s horizontal cross-sectional area (m^2^),
*Ρ* is the air density (kg/m^3^),
*C_D_* is the drag coefficient,

The horizontal force acting on the life-ring in a fluid flow can be expressed as

(8) $$F_{L}=C_{L}F_{w}=C_{D}\frac{1}{2}\rho v_{w}^{2}A_{1}$$

where:

*F_L_* is the horizontal force (*N*),
*C_L_* is the lifting coefficient
*F_w_* is the wind force (*N*)
*A*_1_ is the vertical life-ring’s cross-sectional area (m^2^)

As a random variable, the horizontal force can act on the life-ring in any direction. However, we assumed the worst-case scenario where this force acts on the horizontal direction during the life-ring’s fall down.

Taking into account the UAV’s technical specifications, the maximum allowable wind-speed limit for safe flights is 5Bf (28–39 km/h) and hence we can estimate the life-ring’s maximum horizontal displacement under several horizontal wind speeds. According to Figure 9, in order to stay within the limits of ±0.5 m accuracy, in a worst-case scenario of 40 km/h side-wind, the life-ring must be released from a maximum altitude of 2 m. This can be considered as a safe distance, in the sense that it does not prevent a human in peril from grasping the life preserver.

Finally, the aerial vehicle follows the reverse trajectory and automatically returns to the landing area. Then, the system is prepared for the next rescue mission by its administrator and gets in ready-to-fly state.

## 6. Conclusions

The combination of UAV technologies and real-time computer vision with deep learning techniques for search and rescue purposes has been presented in this work. A novel rescue platform for the instantaneous detection and rescue of humans in peril has been integrated. Based-on state-of-the-art human detection techniques, the proposed system is capable of precisely detecting and rescuing open water swimmers in peril, without apparent human intervention. Distressed swimmer detection is performed on-board a fully autonomous rescue UAV, capable of executing time-crucial life-saving operations in a fully unsupervised manner.

Hardware and software configurations for such implementation have also been outlined. The novelty of this implementation banks on the combination of GNSS techniques and computer vision algorithms. The model used in this research was a CNN with Tiny YOLOv3 architecture running on the embedded autonomous computing machine NVIDIA Jetson X1. Training, validation, and testing processes have also been presented. Lastly, due to the restricted amount of training dataset available online, the authors obtained their own aerial images for the training dataset from the proposed aerial rescue UAV. Real flights evaluation revealed the superiority of the adopted deep learning techniques. Our model’s attained mAP was in the order of 67%, which is a very adequate figure for an on-board human detection process. Moreover, simulation results for the rescue apparatus fall down accuracy are presented. 

The onboard image processing hardware, using the Nvidia Jetson TX1, offers the capability of real-time processing, thus avoiding the transmission of the video sequence to a ground station for processing and returning the results to the UAV, something that could lead to undesirable and crucial time delays.

While the proposed rescue system has been implemented for open water swimmer detection, it would be capable of detecting humans and providing emergency services to people practicing winter sport activities, with only a few modifications. Based on the high level of the achieved accuracy for both detection and classification, there are limitless opportunities for the proposed approach to be applied in SAR missions in numerous terrains or environments.

## Figures and Tables

**Table 1:** Rescue UAV’s technical specifications.

Specifications	
Rotors	6
Weight	2700 g (Excluding Battery)
Brushless Motors	5008-kV 400 (RPM/V)
Motors Max. Current	30 A
Payload Capability	5 Kg
Frame Type	Folding Umbrella
Frame Material	Lightweight Frame, Portable, Carbon Fiber
Landing Gear	Electric Folding
Wheel Base	96 cm
Battery Type	LiPo/22.2 V/16,000 mAh/30 C
Rotor Size	1855 High-end Carbon Fiber
Brushless ESC	40 A/6 S
Autopilot	PX4 (168 MHz, Cortex M4F CPU, 256 KB RAM, 2 MB Flash)
Camera	GoPro 1920 × 1080 HD
Hover Power	1800 W
Hover Time (max)	25 min

## Abbreviations

The following abbreviations are used in this manuscript:

UAVs	Unmanned Aerial Vehicles
SAR	Search and Rescue
GNSS	Global Navigation Satelite System
ROLFER	Robotic Lifeguard For Emergency Rescue
PPP	Precise Point Positioning
CNNs	Convolutional Neural Networks
T-CNNs	Temporal Convolutional Neural Networks
mAP	mean Average Precision
UAS	Unmanned Aerial System
SVM	Support Vector Machine
FCNN	Fully Convolutional Neural Network
IoU	Intersection Over Union
CPU	Central Processing Unit
GPU	Graphics Processing Unit
FPS	Frames Per Second
SP	Supervised Person
GS	Ground Station
ASW	Android Smart Watch
FOV	Field Of View
